# Measuring Hypertension Progression With Transition Probabilities: Estimates From the WHO SAGE Longitudinal Study

**DOI:** 10.3389/fpubh.2021.571110

**Published:** 2021-04-07

**Authors:** Godfred O. Boateng, Stella T. Lartey, Philip Baiden, Lei Si, Richard Berko Biritwum, Paul Kowal, Costan G. Magnussen, Ziyad Ben Taleb, Andrew J. Palmer, Isaac Luginaah

**Affiliations:** ^1^Department of Kinesiology, College of Nursing and Health Innovations, The University of Texas at Arlington, Arlington, TX, United States; ^2^Department of Epidemiology and Biostatistics, School of Public Health-Bloomington, Indiana University School of Public Health-Bloomington, Bloomington, IN, United States; ^3^School of Social Work, The University of Texas at Arlington, Arlington, TX, United States; ^4^The George Institute for Global Health, University of New South Wales, Kensington, NSW, Australia; ^5^Department of Community Health, University of Ghana, Accra, Ghana; ^6^World Health Organization, Geneva, Switzerland; ^7^University of Newcastle Research Centre for Generational Health and Ageing, Newcastle, NSW, Australia; ^8^Menzies Institute for Medical Research, University of Tasmania, Hobart, TAS, Australia; ^9^Research Centre of Applied and Preventive Cardiovascular Medicine, University of Turku, Turku, Finland; ^10^Centre for Population Health Research, University of Turku and Turku University Hospital, Turku, Finland; ^11^Centre for Health Policy, School of Population and Global Health, The University of Melbourne, Melbourne, VIC, Australia; ^12^Department of Geography, University of Western Ontario, London, ON, Canada

**Keywords:** elevated blood pressure, hypertension, multi-state model, transition probabilites, ACC/AHA 2017 hypertension guidelines, sub-Saharan Africa

## Abstract

This paper assessed the transition probabilities between the stages of hypertension severity and the length of time an individual might spend at a particular disease state using the new American College of Cardiology/American Heart Association hypertension blood pressure guidelines. Data for this study were drawn from the Ghana WHO SAGE longitudinal study, with an analytical sample of 1884 across two waves. Using a multistate Markov model, we estimated a seven-year transition probability between normal/elevated blood pressure (systolic ≤ 129 mm Hg & diastolic <80 mm Hg), stage 1 (systolic 130-139 mm Hg & diastolic 80-89 mm Hg), and stage 2 (systolic ≥140mm Hg & diastolic≥90 mm Hg) hypertension and adjusted for the individual effects of anthropometric, lifestyle, and socio-demographic factors. At baseline, 22.5% had stage 1 hypertension and 52.2% had stage 2 hypertension. The estimated seven-year transition probability for the general population was 19.0% (95% CI: 16.4, 21.8) from normal/elevated blood pressure to stage 1 hypertension, 31.6% (95% CI: 27.6, 35.4%) from stage 1 hypertension to stage 2 hypertension, and 48.5% (45.6, 52.1%) for remaining at stage 2. Other factors such as being overweight, obese, female, aged 60+ years, urban residence, low education and high income were associated with an increased probability of remaining at stage 2 hypertension. However, consumption of recommended servings of fruits and vegetables per day was associated with a delay in the onset of stage 1 hypertension and a recovery to normal/elevated blood pressure. This is the first study to show estimated transition probabilities between the stages of hypertension severity across the lifespan in sub-Saharan Africa. The results are important for understanding progression through hypertension severity and can be used in simulating cost-effective models to evaluate policies and the burden of future healthcare.

## Introduction

Uncontrolled hypertension—or elevated blood pressure (BP) is a leading risk factor of cardiovascular diseases and global mortality (1–3). The increase in demographic aging, urbanization, consumption of energy dense and nutrient poor foods, and the globalization of unhealthy lifestyles has increased the risk of hypertension especially, for populations where universal health coverage remains low and inaccessible to many (2). This is most predominant in sub-Saharan Africa (SSA), where the prevalence of hypertension is the highest, affecting 46% of adults older than 25 years compared to 35% in the America's and 40% for the rest of the world (2). This health risk has the potential to decrease the life expectancy of populations in these regions, where majority have no awareness of their hypertensive status (4, 5), and those who are even aware have a lower likelihood of treating and controlling the disease (6). An assessment of the state of hypertension care in 44 Low- and Middle-Income Countries (LMICs) showed that for those with hypertension, 29.9% had received treatment and only 10.3% had achieved control, with worse performances for countries in SSA (7).

To diagnose and control hypertension, the measurement of BP—categorized based on either internationally recognized or arbitrary thresholds—is used in determining the level of severity and the required treatment in cases of values with increased risk of cardiovascular diseases (CVD) (8). The American College of Cardiology/American Heart Association (ACC/AHA) recently developed a new guideline for the prevention, detection, evaluation, and management of high BP among adults in the United States (9). This guideline is an updated version of the Seventh report of the Joint National Committee (JNC-7) on Prevention, Detection, Evaluation, and Treatment of high BP published in 2003 and aimed at improving hypertension treatment and control (10). The new guideline defines normal BP as Systolic BP <1 20/Diastolic BP <80 mm Hg; elevated BP as 120–129/ <80 mm Hg; stage 1 hypertension as 130–139 or 80–89 mm Hg, and stage 2 hypertension as ≥ 140 or ≥90 mm Hg (9, 11). These new cut-offs relative to the 2003 version (10) are touted to increase the number of people diagnosed as having hypertension in each age group (12). Although critiqued for the psychosocial effects it could have on previously healthy people who will now be labeled as hypertensive and the potential to increase financial burden on households and governments (12), the new guidelines have the potential of detecting early, elevated BP or Stage 1 hypertension which have been found to increase the likelihood of CVD (13). While its applicability to developing countries has been questioned (12), the urgent need for early detection, treatment, and control of hypertension with the potential to decrease cardiometabolic diseases and mortality makes its application warranted. Untreated hypertension could open the gateway to all kinds of CVDs, which are now on the increase in LMICs (3, 14, 15).

The need to control and manage the risk of hypertension has become even more critical due to the detrimental consequences it has on the individual, families, communities, and governments at large. At the individual level, it leads to a deterioration of health, exposure to several diseases and health risks such as ischemic heart disease, stroke, blindness, rupture of blood vessels, cognitive impairment, arrhythmias, vascular dementia, and kidney failure (2, 16). Households carry much of the physical, emotional, and financial burden when family members experience complications with hypertension. For some, this financial drain has the potential to lead to a decline in household income and increase impoverishment (17). Within the community, it could lead to both, human capital and financial losses (18). At the national level, this has the potential to escalate healthcare expenditure putting the management of other critical diseases at a deficit and could also stifle economic development. Additionally, underdeveloped health systems in regions such as SSA compound the problem as several people with hypertension may go undiagnosed, untreated, and uncontrolled thereby increasing the burden of diseases and risk of mortality (2, 19).

To better manage and target policies and resources toward the prevention of hypertension, it is useful to understand the probabilities associated with transitioning from one stage of this medical condition to the other. Knowledge of the burden associated with this health risk and its management has the potential to improve medical and financial decisions aimed at reducing mortality associated with hypertension. Thus, knowing how long it takes a population to transition from normal BP to elevated BP, or stage 1 or stage 2 hypertension is vital for public health and government fiscal policies and planning. Therefore, this study aimed at estimating the transition probabilities between the different stages of hypertension severity (normal/elevated BP, stage 1 hypertension, and stage 2 hypertension) over a seven-year period.

Previous studies on LMICs have tried to estimate the relationships between the stages of hypertension and associated factors using random effects multinomial logistic regression (18). However, such studies do not estimate sojourn times at a disease state or transition probabilities to a worse state or recovery to a healthy state. The use of multistate models presents a novel statistical approach in analyzing the progression between the different stages/states of hypertension severity as used in previous studies to describe the complexities of diseases involving multiple states (20–22).

Although several multistate models have been used to estimate transition probabilities (23), we use the Markov model, which describes the processes by which individuals progress through a finite number of health states in a unit of time (24), to estimate the transition probabilities between three distinct states of hypertension. We hypothesize that:

1. The transition probability in the general population from normal/elevated BP to stage 1 hypertension will be higher than the transition probability to stage 2 hypertension over a 7-year period.
2. The probability of reverting back to normal/elevated BP from stage 1 will be higher than transitioning to stage 2 hypertension.
3. The probability of staying at stage 2 will be higher than the probability of staying at stage 1 over the 7-year period.
4. The transition probability to a more severe hypertensive state will be higher for obese participants than those with normal weight over a 7-year period.

## Materials and Methods

### Study Population

We used data from the World Health Organization Study on global AGEing and adult health (WHO SAGE) for this study. The WHO SAGE is a longitudinal study on the health and well-being of adult populations aged ≥50 years in six countries: China, Ghana, India, Mexico, Russian Federation, and South Africa. It also collects sample data of younger adults, aged 18–49 years for comparison (25). For this study, a combination of data from both the younger and older cohorts were used. In Ghana, SAGE collected individual-level data from nationally representative households of adults using stratified, multistage cluster design. Primary sampling units were stratified by region and locality (rural/urban) at baseline and the same was used at waves 1 (2007/2008) and 2 (2014/2015). At wave 1 and wave 2, 5,110 and 4,704 respondents, respectively, aged 18 years and older participated in the study. However, an analytical sample of 1,884 was used for those who participated in wave 1 and 2, after the exemption of pregnant women. The SAGE data is unique because of the objective measurements made on hypertension, anthropometric measurement on Body-Mass Index (BMI), and socio-demographic characteristics which are covered in the individual survey. Systematic replacement was used in Wave 2 to account for losses to the sample from Wave 1.

### Classification of Hypertension Stages

The measure of hypertension was taken from objective measurements of respondents' BP (systolic/diastolic), which were taken 3 times. Measurements were conducted using a Boso Medistar Wrist Blood Pressure Monitor Model S. To take the readings, respondents were asked to sit quietly with their legs uncrossed and relaxed (26). The wrist cuff of the device was then wrapped around the respondent's left wrist and secured with the Velcro strap for a snug fit. The respondent was then made to place the wrist against their chest approximately at the level of their heart (27). Additionally, the hand was made to rest approximately at the level of the shoulder, the elbow at the waist, and the arm against the body. The free hand was then placed under the elbow to support the arm with the device on the wrist. The respondent was asked to take 3 deep, slow breaths before the measurement started. With each measurement, the staff recorded the systolic BP, diastolic BP, and pulse rate. After each recording, the respondent rested for a minute until the next reading for all three recordings (27).

The mean of all three recordings was used as a diagnosis for scores on BP, which was normally distributed and then categorized based on the new guidelines by the ACC/AHA (9). The ACC/AHA recognize four BP groups in adults: Normal BP (SBP <120 and DBP <80 mmHg); elevated BP (120–129 & <80 mm Hg); stage 1 hypertension (130–139/80–89 mm Hg), and stage 2 hypertension (≥140/≥90 mm Hg)(9, 11). At stage 1, lifestyle and behavioral changes including tobacco cessation, weight loss, moderation in alcohol intake, increased physical activity, reduced sodium intake, and consumption of a healthy diet are recommended. Adults are encouraged to adhere to these changes over a long period to maximize the benefit of a healthy lifestyle (9). At stage 2, doctors will prescribe a combination of BP medication and lifestyle changes. In order to assess the transition probability between extreme forms of hypertension, we merged normal and elevated BP to create three categories instead of four. Hypertension stages were differentiated into normal/elevated BP (SBP ≤ 129 and DBP <80 mmHg); stage 1 hypertension (SBP:130–139/DBP: 80–89 mm Hg), and stage 2 hypertension (SBP≥140/DBP ≥ 90 mm Hg).

### Classification of Body Mass Index

Respondents were measured for their body weight and height by trained assessors using standard protocol (27). Pregnant women were exempted from weight measurements in both surveys (27). BMI was calculated as the person's weight in kilograms divided by the square of their height in meters (kg/m^2^). WHO classifications were used to categorize BMI as follows: normal/healthy weight, BMI ≥18.5 and <25.0 kg/m^2^; underweight, BMI <18.5; overweight, BMI = 25.0 and <30.0 kg/m^2^; and obesity as BMI ≥30.0 kg/m^2^ (28).

#### Other Covariates

Other covariates used in this study included sex, coded as “1= female” vs. “2= male;” age, coded as “1= ≤ 40 years,” “2 = 41–60 years,” and “3 = above 60 years;” and level of education which was coded as “1 = less than high school” vs. “2 = high school and above.” Place of residence was coded as “1 = rural” vs. “2 = urban;” household wealth index constructed from a total of 22 assets/ characteristics/ items were converted into wealth quintiles (lowest, low, moderate, high, higher) (29, 30); and fruits and vegetables intake per day if they consumed ≥5 servings of fruits and/or vegetables per day (equivalent to 400 g), coded as “1 = met requirement” and “2 = below requirement” according to international standards (31). Even though self-reported medication or therapy over the past 12 months is reported in the summary statistics, this was not used in the model because of 90% (Wave 1) and 99% (Wave 2) missing reported data.

### Statistical Analysis: Calculation of Transition Probabilities

**Figure 1:**
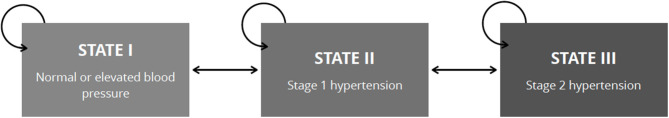
Allowable transitions between hypertension severity states used to estimate 7-year transition probabilities. The arrows illustrate the possible transitions between states. State transition can be a transition into the immediate next state, previous or into itself. A bidirectional transition is allowed. A two-step movement is allowed; however, the subject may pass through the immediate adjacent state first. Respondent can be in only one state at a time.

We conducted descriptive analysis of the sample. Next, we estimated a multistate Markov model (24) as described elsewhere (32). Seven-year transition probabilities from the cohort were estimated using a validated multistate continuous-time Markov model in the “msm” package in R (24). A transition probability meets the requirement of the Markov assumption that the conditional distribution of future states of the process given current and past states depends only on the current state and not on the past states (24). The Markov model has been applied and validated in several previous clinical studies (21, 22). In this study, we applied a three-hypertension-states model with states labeled as normal and elevated, stage 1 and stage 2 (Figure 1). In this model, the underlying progression into a specific state rather than the observed progression is emphasized since transition may have mostly occurred outside the follow-up dates and a two-step movement (i.e., movement into the next two states) is not necessarily instantaneous. Thus, a person with normal or elevated BP at time *t* = 0, may pass through the stage 1 hypertension state before ending in the stage 2 hypertension at time *t* = 7.

Since the exact time of transition is not observed but evolved continuously in time, and is presumed to have occurred before the next assessment, the state-to-state transitions are interval-censored (21, 22, 24). Thus, due to unknown exact transition time and irregular follow-up times for assessment among individual observations, the standard multistate model could not be used. Rather, we employed the time-homogenous continuous-time Markov model that can accommodate such irregularities to calculate transition intensities and the transition probabilities (24). At time *t* in the model, the individual is in state *S(t)*. Movement into the next state is guided by a set of transition intensities, (i.e., the rate at which an individual has the likelihood of moving from one state *i* to another state *j* at a given time (*t*). The intensities form a “3 × 3” matrix *Q* whose rows sum to zero. The diagonals of the matrix are the rates at which individuals do not transition and remain in their state whereas the off-diagonals represent the rates at which individuals transition into other states (21, 22, 24). The model specified that an individual transitioning to a nonadjacent state (e.g., from normal/elevated BP to stage 2 hypertension) must have passed through the immediate adjacent state (i.e., stage 1 hypertension state) rather than a direct movement to a nonadjacent state. Therefore, the maximum likelihood estimate was zero for the nonadjacent states (i.e., a two-step movement).

Under the assumption that *Q* is constant over time, the transition probability *P*(*t*), which is the chance of transitioning from one stage to the other is calculated by taking the matrix exponential of the scaled transition intensity matrix as follows:

P(t)=Exp(tQ)t ≥ 0.

In this study, a 7-year transition probability was calculated. Additionally, using the unadjusted model, we calculated the mean sojourn times (i.e., the average time spent in a specific state before transitioning into another state) for each state. Due to the model flexibility, covariates including sex, age, educational level, place of residence and household wealth status were fitted in separate models to examine if these factors changed the probabilities.

To validate the estimated transition probabilities, a Markov model was developed in TreeAge Pro 2018 (TreeAge Software Inc. Williamstown, Massachusetts, USA). Four Markov states were included in the model, that is, normal or elevated BP, stage 1 and stage 2 hypertension and death. Simulated subjects' transit between states using the calculated transition probabilities. Age-specific mortality risk was taken from the Ghanaian life table (33). A 5-year interval cohort analysis and prevalence of the three hypertension states were compared to the observed prevalence in Wave 2. The probabilities were valid if the observed prevalence in Wave 2 fell within the 95% CI of the estimated expected prevalence or if the observed prevalence in Wave 2 falls 1% lower or higher above the expected prevalence.

Statistical analyses were performed using R (R Foundation for Statistical Computing, Vienna, Austria) and validation was assessed using TreeAge Pro 2018.

## Results

The sample characteristics are shown in Table 1. An analytical sample of 1,884 respondents was examined at both time points. Of this sample, 25.3% had normal/elevated BP, 22.5% could be classified as having stage 1 hypertension, 52.2% as stage 2 hypertension at wave 1. By wave 2, 42.5% could be classified as having normal/elevated BP, 21.1% at stage 1 hypertension, 36.4% at stage 2 hypertension. The sex distribution was the same across both time points, with males constituting 56% and females 44%. By wave 2, 2.7% were <40 years, 29.1% were between 41–60 years, and 68.2% were more than 60 years old. Of the 1,884 respondents, 13.4% were underweight, 55.1% had normal weight, 20.4% were overweight, and 11.1% were obese.

**Table 1:** Descriptive statistics of variables used in assessing progression in hypertension severity between 2007/2008 to 2014/2015.

**Variables**	**Wave 1 (2007/8)**	**Wave 2 (2014/15)**
	***n* (%)**	***n* (%)**
Sample size	1884	1884
**Hypertension stages**		
Normal/elevated BP	477 (25.3)	801 (42.5)
Stage 1	424 (22.5)	397 (21.1)
Stage 2	983 (52.2)	686 (36.4)
**Sex**		
Males	1055 (56.0)	1055 (56.0)
Females	829 (44.0)	829 (44.0)
**Age group, years**		
≤ 40	97 (5.2)	51 (2.7)
41-60	942 (50.0)	548 (29.1)
>60	845 (44.9)	1285 (68.2)
**BMI categories**		
Underweight	237 (12.6)	252 (13.4)
Normal weight	1112 (59.0)	1038 (55.1)
Overweight	364 (19.3)	385 (20.4)
Obesity	171 (9.1)	209 (11.1)
**Educational status**		
High	505 (26.8)	514 (27.3)
Low	1379 (73.2)	1370 (72.7)
**Place of residence**		
Rural	1160 (61.9)	1167 (61.9)
Urban	724 (38.1)	717 (38.1)
**Wealth index**		
Lowest	339 (18.0)	237 (12.6)
Low	397 (21.1)	463 (24.6)
Moderate	422 (22.4)	401 (21.3)
High	391 (20.8)	419 (22.2)
Higher	335 (17.8)	364 (19.3)
**Medications or other treatment in the past 12 months**		
No response	1695 (90)	1,866 (99)
No	40 (2.1)	8 (0.4)
Yes	149 (7.9)	10 (5)
**Fruit and vegetable intake**		
Below requirement	1,316 (69.9)	832 (44.2)
Met requirement	568 (30.2)	1,052 (55.8)

*BP, Blood Pressure; (%), percentages*.

We show the prevalence (95% CI) of the three hypertension stages in 2007/2008 and 2014/2015 in Table 2. While the prevalence of normal BP increased, stage 1 and 2 hypertension decreased in wave 2.

**Table 2:** Prevalence (95% CI) of hypertension severity between 2007/2008 to 2014/2015.

		**Wave 1**	**Wave 2**
		**Prevalence**	**Prevalences**
		**(95% CI)**	**(95% CI)**
n		1884	1,884
Hypertension Stages	Normal/elevated BP	25.3 (23.4, 27.3)	42.5 (40.3, 44.8)
	Stage 1 Hypertension	22.5 (20.7, 24.4)	21.1 (19.3, 23.0)
	Stage 2 Hypertension	52.1 (49.9, 54.4)	36.4 (34.3, 38.6)

*Wave 1 = 2007/2008; Wave 2 =2014/2015*.

Table 3 shows the frequency of transition between the three hypertension stages from 2007/2008 at the beginning to 2014/2015 at the end. Transitions occurred between all three stages observed in this study. Ninety-three transitioned from normal BP to stage 1 hypertension, with 80 transitioning from normal BP to stage 2 hypertension over the 7-year period. One hundred and ninety-nine transitioned from stage 1 hypertension to normal /elevated BP, 89 remained at stage 1, while 136 progressed to stage 2 hypertension. Two hundred and fifteen transitioned from stage 2 hypertension to stage 1,298 from stage 2 to normal/elevated BP, while 470 remained at stage 2 hypertension.

**Table 3:** Total number of transitions between three hypertension states from 2007/2008 to 2014/2015.

	**To:**		
**From: wave 1**	**Wave 2**		
**Hypertension severity**	**Normal/elevated blood pressure**	**Stage 1 hypertension**	**Stage 2 hypertension**
Normal/elevated BP	304	93	80
Stage 1 hypertension	199	89	136
Stage 2 hypertension	298	215	470

*Wave 1 = 2007/2008; Wave 2 =2014/2015*.

Table 4 shows the transition intensity matrix of the maximum likelihood estimates (95% CI) over a 7-year period. The results do show a likely transition from normal/elevated BP to stage 1 hypertension (0.129) and a recovery from stage 2 hypertension to stage 1 hypertension (0.185); however, this likelihood increased for a transition from stage 1 hypertension to normal BP/elevated BP (0.321) and stage 1 hypertension to stage 2 hypertension (0.264). The mean sojourn time from normal/elevated BP to stage 1 hypertension was −1/ (−0.129) =7.75 years, −1/ (−0.584) = 1.7 years for stage 1 hypertension to stage 2 hypertension or recover back to normal/elevated BP, and −1(−0.185) = 5.4 years from stage 2 hypertension to stage 1 hypertension.

**Table 4:** Transition intensity matrix for a Multistate Markov Model of the transition between normal/elevated blood pressure, Stage 1 and Stage 2 hypertension across adults (18+ years) in Ghana between 2007/2008-2014/2015.

	**To**					
**From**	**Normal/elevated BP**		**Stage 1 Hypertension**		**Stage 2 Hypertension**	
Hypertension state	MLE^a^	95% CI	MLE	95% CI	MLE	95% CI
Normal/elevated BP	−0.129	−0.167, −0.100	0.129	0.100, 0.167	0^b^	–
Stage 1 hypertension	0.321	0.260, 0.395	−0.584	−0.678, −0.504	0.264	0.203, 0.343
Stage 2 hypertension	0^b^	–	0.185	0.152, 0.225	−0.185	0.225, −0.152

*BP, Blood Pressure; CI, Confidence intervals; MLE, Maximum Likelihood Estimates; the mean time spent in the Normal/elevated BP state before a transition was made into another state was −1/ (−0.129) =7.8 years, a mean of −1/ (−0.584) =1.7 years for State 1 Hypertension, and a mean of −1/ (−0.185) =5.4 years for State 2 Hypertension*;

a *MLE, maximum likelihood estimate*;

b *MLE was zero for the nonadjacent states*.

Using the information from the two waves, the 7-year transition probabilities were estimated and are shown in Table 5. The 7-year transition probability from normal/elevated BP to stage 1 hypertension was 19% (95% CI: 16.4, 21.8%) and from normal/elevated BP to stage 2 hypertension was 16.9% (95% CI: 14.1, 19.6%). After 7 years, the probability of transitioning from stage 1 hypertension to normal/elevated BP was 47.3% (95% CI: 43.6%, 51.2%) and stage 1 hypertension to stage 2 hypertension was 31.6% (95% CI: 27.6, 35.4%). For respondents who were at stage 2 hypertension 7 years ago, the probability of transitioning to stage 1 hypertension was 22.1% (95% CI: 19.9, 24.5%), reverting to normal BP/elevated BP was 29.4% (95% CI: 26.7, 31.8%), and remaining at stage 2 hypertension was 48.5% (95% CI: 45.6, 52.1%).

**Table 5:** Seven-year transition probabilities between hypertension severity states calculated from Ghana WHO-SAGE Waves 1 and 2 for the general population and subgroups.

			**To**		
		**From**	**Normal Blood Pressure**	**Stage 1 Hypertension**	**Stage 2 Hypertension**
			**Prob. (95% CI)**	**Prob. (95% CI)**	**Prob. (95% CI)**
General population	Hypertension states	Normal/elevated Blood Pressure	0.641 (0.600, 0.683)	0.190 (0.164, 0.218)	0.169 (0.141, 0.196)
		Stage 1 Hypertension	0.473 (0.436, 0.512)	0.211 (0.192, 0.233)	0.316 (0.276, 0.354)
		Stage 2 Hypertension	0.294 (0.267, 0.318)	0.221 (0.199, 0.245)	0.485 (0.456, 0.521)
Sex	Male	Normal/elevated Blood Pressure	0.653 (0.601, 0.706)	0.200 (0.165, 0.234)	0.147 (0.116, 0.185)
		Stage 1 Hypertension	0.496 (0.445, 0.538)	0.222 (0.197, 0.253)	0.282 (0.237, 0.327)
		Stage 2 Hypertension	0.302 (0.267, 0.337)	0.233 (0.204, 0.267)	0.465 (0.422, 0.506)
	Female	Normal/elevated Blood Pressure	0.622 (0.551, 0.694)	0.176 (0.137, 0.221)	0.203 (0.158, 0.250)
		Stage 1 Hypertension	0.438 (0.378, 0.495)	0.196 (0.172, 0.228)	0.366 (0.302, 0.429)
		Stage 2 Hypertension	0.287 (0.246, 0.319)	0.208 (0.177, 0.240)	0.506 (0.465, 0.558)
Age group, years	≤ 40	Normal/elevated Blood Pressure	0.739 (0.626, 0.899)	0.161 (0.063, 0.253)	0.100 (0.028, 0.185)
		Stage 1 Hypertension	0.705 (0.342, 0.788)	0.159 (0.100, 0.356)	0.136 (0.065, 0.355)
		Stage 2 Hypertension	0.455 (0.168, 0.596)	0.141 (0.076, 0.377)	0.404 (0.255, 0.607)
	41-60	Normal/elevated Blood Pressure	0.648 (0.592, 0.708)	0.196 (0.157, 0.236)	0.156 (0.119, 0.194)
		Stage 1 Hypertension	0.417 (0.415, 0.518)	0.230 (0.204, 0.260)	0.299 (0.250, 0.351)
		Stage 2 Hypertension	0.313 (0.275, 0.353)	0.250 (0.217, 0.280)	0.437 (0.398, 0.486)
	>60	Normal/elevated Blood Pressure	0.616 (0.551, 0.684)	0.184 (0.144, 0.227)	0.200 (0.157, 0.245)
		Stage 1 Hypertension	0.442 (0.385, 0.493)	0.195 (0.169, 0.229)	0.363 (0.306, 0.418)
		Stage 2 Hypertension	0.262 (0.223, 0.297)	0.198 (0.169, 0.230)	0.539 (0.496, 0.587)
BMI categories	Underweight	Normal/elevated Blood Pressure	0.615 (0.524, 0.763)	0.218 (0.129, 0.291)	0.167 (0.091, 0.230)
		Stage 1 Hypertension	0.522 (0.352,0.610)	0.222 (0.174, 0.305)	0.257 (0.163, 0.382)
		Stage 2 Hypertension	0.342 (0.224, 0.421)	0.221 (0.147, 0.319)	0.437 (0.354, 0.563)
	Normal weight	Normal/elevated Blood Pressure	0.638 (0.587, 0.689)	0.201 (0.167, 0.235)	0.161 (0.129, 0.1932)
		Stage 1 Hypertension	0.471 (0.419, 0.519)	0.225 (0.199, 0.253)	0.305 (0.258, 0.356)
		Stage 2 Hypertension	0.286 (0.253, 0.319)	0.232 (0.205, 0.262)	0.481 (0.443, 0.526)
	Overweight	Normal/elevated Blood Pressure	0.646 (0.529, 0.759)	0.154 (0.097, 0.220)	0.200 (0.123, 0.282)
		Stage 1 Hypertension	0.467 (0.382, 0.547)	0.175 (0.142, 0.226)	0.358 (0.270, 0.443)
		Stage 2 Hypertension	0.323 (0.254, 0.379)	0.191 (0.149, 0.239)	0.486 (0.432, 0.569)
	Obesity	Normal/elevated Blood Pressure	0.722 (0.493, 0.870)	0.125 (0.048, 0.250)	0.153 (0.060, 0.308)
		Stage 1 Hypertension	0.399 (0.260, 0.544)	0.186 (0.135, 0.272)	0.415 (0.254, 0.553)
		Stage 2 Hypertension	0.246 (0.159, 0.304)	0.209 (0.144, 0.282)	0.546 (0.472, 0.668)
Educational status	High	Normal/elevated Blood Pressure	0.692 (0.613, 0.763)	0.168 (0.124, 0.223)	0.140 (0.097, 0.189)
		Stage 1 Hypertension	0.454 (0.379, 0.525)	0.221 (0.184, 0.267)	0.325 (0.2595, 0.393)
		Stage 2 Hypertension	0.286 (0.236, 0.332)	0.246 (0.203, 0.289)	0.468 (0.415, 0.534)
	Low	Normal/elevated Blood Pressure	0.623 (0.573, 0.672)	0.197 (0.168, 0.229)	0.180 (0.147, 0.214)
		Stage 1 Hypertension	0.480 (0.437, 0.518)	0.208 (0.188, 0.233)	0.312 (0.268, 0.357)
		Stage 2 Hypertension	0.297 (0.264, 0.326)	0.212 (0.190, 0.239)	0.491 (0.458, 0.531)
Place of residence	Rural	Normal/elevated Blood Pressure	0.644 (0.593, 0.695)	0.194 (0.161, 0.228)	0.163 (0.133, 0.196)
		Stage 1 Hypertension	0.493 (0.443, 0.535)	0.211 (0.189, 0.240)	0.296 (0.252, 0.343)
		Stage 2 Hypertension	0.309 (0.270, 0.343)	0.221 (0.193, 0.252)	0.471 (0.431, 0.516)
	Urban	Normal/elevated Blood Pressure	0.635 (0.556, 0.708)	0.183 (0.142, 0.236)	0.182 (0.132, 0.231)
		Stage 1 Hypertension	0.439 (0.378, 0.497)	0.209 (0.181, 0.247)	0.352 (0.285, 0.414)
		Stage 2 Hypertension	0.275 (0.232, 0.311)	0.222 (0.189, 0.257)	0.503 (0.460, 0.560)
Wealth index	Lowest	Normal/elevated Blood Pressure	0.601 (0.526, 0.686)	0.207 (0.153, 0.263)	0.192 (0.133, 0.251)
		Stage 1 Hypertension	0.490 (0.386, 0.564)	0.209 (0.171, 0.263)	0.301 (0.221, 0.387)
		Stage 2 Hypertension	0.309 (0.234, 0.374)	0.205 (0.153, 0.271)	0.486 (0.408, 0.580)
	Low	Normal/elevated Blood Pressure	0.658 (0.565, 0.745)	0.205 (0.145, 0.268)	0.137 (0.090, 0.199)
		Stage 1 Hypertension	0.513 (0.421, 0.588)	0.220 (0.179, 0.279)	0.267 (0.193, 0.359)
		Stage 2 Hypertension	0.278 (0.215, 0.331)	0.216 (0.171, 0.271)	0.506 (0.437, 0.581)
	Moderate	Normal/elevated Blood Pressure	0.668 (0.574, 0.754)	0.195 (0.137, 0.262)	0.137 (0.088, 0.194)
		Stage 1 Hypertension	0.501 (0.421, 0.568)	0.225 (0.188, 0.280)	0.274 (0.198, 0.360)
		Stage 2 Hypertension	0.306 (0.247, 0.356)	0.239 (0.195, 0.292)	0.455 (0.399, 0.525)
	High	Normal/elevated Blood Pressure	0.645 (0.553, 0.736)	0.173 (0.119, 0.235)	0.182 (0.123, 0.250)
		Stage 1 Hypertension	0.489 (0.399, 0.562)	0.183 (0.148, 0.239)	0.328 (0.244, 0.416)
		Stage 2 Hypertension	0.296 (0.233, 0.347)	0.189 (0.150, 0.239)	0.515 (0.460, 0.592)
	Higher	Normal/elevated Blood Pressure	0.639 (0.520, 0.746)	0.161 (0.105, 0.232)	0.200 (0.128, 0.287)
		Stage 1 Hypertension	0.371 (0.288, 0.468)	0.225 (0.186, 0.281)	0.404 (0.312, 0.480)
		Stage 2 Hypertension	0.280 (0.209, 0.329)	0.245 (0.196, 0.297)	0.474 (0.415, 0.575)
Fruit and vegetable intake	Met requirement	Normal/elevated Blood Pressure	0.908 (0.864, 0.940)	0.081 (0.051, 0.122)	0.012 (0.007, 0.020)
		Stage 1 Hypertension	0.241 (0.177, 0.317)	0.571 (0.484, 0.641)	0.188 (0.124, 0.281)
		Stage 2 Hypertension	0.025 (0.018, 0.035)	0.134 (0.099, 0.177)	0.841 (0.794, 0.881)
	Below requirement	Normal/elevated Blood Pressure	0.890 (0.856, 0.916)	0.096 (0.072, 0.128)	0.013 (0.010, 0.018)
		Stage 1 Hypertension	0.223 (0.181, 0.273)	0.595 (0.540, 0.640)	0.182 (0.141, 0.232)
		Stage 2 Hypertension	0.021 (0.017, 0.026)	0.126 (0.105, 0.154)	0.853 (0.823, 0.876)

*Wave 1: 2007/2008; wave 2: 2014/2015*.

The validity of our calculated transition probabilities was assessed to be good. The observed prevalence for normal/elevated BP, stage 1 hypertension, and stage 2 hypertension at wave 2 was 42.5, 21.1, and 36.1% respectively. The calculated prevalence from the Markov models for normal/elevated BP was 42.2% (95% CI: 41.3, 42.9%), stage 1 hypertension was 21.1% (95%CI: 21.0, 21.3%), and stage 2 hypertension was 36.7% (95% CI: 35.7, 37.7%). All calculated prevalence estimates for hypertension severity fell within the 95% CI or were 1% lower than the observed prevalence of the three states of hypertension severity.

The results for the explanatory variables show an increased transition probability from normal/elevated BP to stage 1 hypertension and stage 1 hypertension. Over a 7-year period, being a male increased the transition probability from normal/elevated BP to stage 1 hypertension (20%; 95% CI: 16.5, 23.4%), stage 1 to stage 2 hypertension (28.2%; 95% CI: 23.7, 32.7%), and remaining at stage 2 hypertension (46.5%, 95% CI: 42.2, 50.6%). Comparatively, females had a higher transition probability from stage 1 to stage 2 hypertension (females: 36.6%; males: 28.2%) and for remaining at stage 2 hypertension (females: 50.6%; males: 46.5%). Between the three age cohorts examined in this study, the transition probability from normal/elevated BP to stage 1 hypertension (19.6%; 95% CI:15.7, 23.6%) and from stage 1 to stage 2 hypertension (36.3%, 95% CI: 30.6, 21.8%) was higher for those aged 41–60 years and those above 60 years, respectively. However, the probability of remaining at stage 2 hypertension was consistently higher for all age groups (≤ 40: 40.4%; 41–60: 43.7%; >60: 53.9%) but highest among those above 60 years. For BMI categories, the transition probability from normal/elevated BP to stage 1 hypertension increased most for those underweight (21.8%, 95% CI: 12.9, 29.1%) compared to normal weight, overweight, and obesity. However, the probability of transitioning from stage 1 to stage 2 hypertension was much higher among those overweight (35.8%) and obese (41.5%). The transition probability of remaining at stage 2 hypertension was consistently higher across all four BMI cohorts (underweight: 43.7%; normal weight: 48.1%; overweight: 48.6%; obesity: 54.6%), but highest among the obese. Having less than high school education relative to high school or more education (19.7 vs.16.8%), rural relative to urban residence (19.2 vs. 18.3%), lowest wealth quintile relative to higher wealth (20.7 vs.16.1%) increased the transition probability from normal/elevated BP to stage 1 hypertension. Differently, high relative to low education (32.5 vs. 31.2%), urban relative to rural residence (35.2 vs. 29.6%), higher relative to low wealth quintile (40.4 vs. 26.7%) were associated with a higher transition probability from stage 1 to stage 2 hypertension. The transition probability of those who met the WHO recommendation for the intake of fruit and vegetables was 90.8% for remaining at normal/elevated BP, 8.1% for transitioning to stage 1, and 1.2% for transitioning to stage 2. The transition probability back from stage 1 to normal/elevated BP was 90.8% but 18.8% to stage 2 hypertension. Comparatively, those who did not meet the recommended servings of fruits and vegetables had a transition probability of 89% for remaining at normal/elevated BP, 9.6% to transition to stage 1, and 1.3% to stage 2. However, the transition probability back to normal/elevated BP from stage 1 hypertension was 22.3% and 18.2% to stage 2 hypertension.

The transition probability of remaining at stage 2 hypertension was consistently higher for those with both low and high education (49.1 and 46.8%), rural and urban residence (47.1 and 50.3%), lowest, low, moderate, high, and higher wealth quintile (48.6, 50.6, 45.5, 51.5, and 47.4%), and those who met the WHO recommendation for the intake of fruits and vegetables and those who did not meet the requirement (84.1 and 85.3%).

The hazard ratios Appendix A in Supplementary Material were estimated to determine the vector of covariates that were significant predictors of either forward and backward transitions.

## Discussion

This study estimated the transition probabilities between the stages of hypertension severity—normal and elevated BP, stage 1 hypertension, and stage 2 hypertension—and the length of time an individual might spend at a particular disease state. With hypertension being a leading risk factor of CVD and worldwide mortality (1–3), the ability to estimate how long a population stays at a particular health state and when the affected population would either transition to a better or worse state is critical for government and healthcare fiscal policies and planning. Our results show that after a 7-year period, one in five of the general population had a probability of transitioning from normal/elevated BP to stage 1 hypertension; three in ten progressed from stage 1 to stage 2 hypertension; and almost one in two remained at stage 2 hypertension. The novel approach used in this study makes it the first in SSA to examine progression in hypertension states, which has implications for prevalence control, government health expenditure, and cost-effective interventions in managing this medical condition. By the application of the ACC/AHA new guidelines for the prevention, detection, evaluation, and management of high BP in adults, this study shows the importance of having a guideline that will at least facilitate the early detection of hypertensive cases, with the potential for control and treatment. In testing our hypotheses, several important findings were made in this study which are worth discussing and do contribute to the literature on hypertension.

First, over a 7-year period, the probability of progressing from normal/elevated BP to an adjacent state, that is, stage 1 hypertension was 19%, greater than progressing to a non-adjacent state, stage 2 hypertension which was 16.9%. Since an individual can be at a single state at a particular time point (24), the probabilities for stage 1 and stage 2 hypertension are indicative of the high risk of potential disease outcomes and the consistency of our findings with more recent studies showing an increase in the prevalence of hypertension (34).

Second, the probability of recovering from stage 1 hypertension to normal/elevated BP (47.3%) was greater than progressing to stage 2 hypertension (31.6%). Thus, more people recovered to a better health state than those who progressed to a worse health state. This could be due to lifestyle changes, the effectiveness of community based interventions (35), or improved access to hypertension services (36). However, once at stage 2, the probability of remaining at that state even after 7 years was much higher for every one out of two persons, than recovering back to either stage 1 hypertension or even normal/elevated BP. At this stage, the risk of stroke, myocardial infarction, kidney failure, diabetes, and the sequelae of CVD is very high (37) requiring treatment with medication and lifestyle changes including eating a heart-healthy diet with less salt, engaging in regular physical activity, maintaining a healthy weight or losing weight if overweight or obese, and limiting the intake of alcohol to avoid the compounding effect of mortality. However, these changes come at a price to patients, who may not have the finances to consistently buy these medications or the ability to engage in regular physical activity due to functional disabilities and old age. Additionally, the onset of comorbidities may further delay the recovery process.

Again, with a higher likelihood of remaining at stage 2, the financial burden associated with treatment and control is much higher suggesting an increase in healthcare expenditure for governments and out-of-pocket payments for individuals. These facts call for urgent policies and interventions aimed at the prevention of transitions to a worse state and health expenditure projects targeted at treating stage 2 hypertensive populations.

A significant finding of this study is the importance of the new thresholds developed by the ACC/AHA in early detection, control, and treatment of this medical condition (9). Using the current thresholds, we now know that it takes an average of 7 years 10 months to transition from normal/elevated BP to stage 1 hypertension, 1 year 8 months to transition from stage 1 hypertension to either stage 2 hypertension or recover to normal/elevated BP, and 5 years 5 months to transition from stage 2 hypertension to stage 1 hypertension. This provides evidence of the time-window at which awareness and systematic screening can occur to forestall any disease progression, with knowledge that early detection of elevated BP makes it amenable to control and treatment (38), which is effective in reducing the incidence of mortality. Additionally, based on the new ACC/AHA hypertension thresholds, our study provides new transition probabilities for researchers who aim at predicting the risk, evaluating the financial burden, or patient directed care associated with hypertension.

Fourth, to succeed at the implementation of hypertension prevention interventions, knowledge of who to screen plays a key role. In this study, we made important discoveries on various subgroups within the general population. Males had a higher transition probability from normal/elevated BP to stage 1 hypertension; while females compared to males, had a higher transition probability from stage 1 to stage 2 and remaining there. This finding is consistent with previous studies which show that females are more at risk of more severe stages of hypertension than males (18, 39). Similar evidence among older adults; that is, those 60 years and above can also be confirmed in this study.

Previous studies have found a significant association between higher scores of BMI and hypertension. For instance, Boateng et al. (18) found that both overweight and obesity were associated with an increased risk of stage 1 and stage 2 hypertension relative to normal/prehypertension in Ghana. Additional evidence from a systematic review and meta-analysis on hypertension among older adults in Africa (39) did show that overweight and obesity were independently associated with hypertension. The results of our study do not only confirm these relationships but go a step further to show that overweight and obese populations have a higher probability of progressing from stage 1 to stage 2 hypertension, with a lower likelihood of recovery to normal/elevated BP. This could be attributed to a number of factors as obesity stimulates the activation of the renin-angiotensin-aldosterone system, an increase in sympathetic activity, which further promotes insulin and leptin resistance, and an increase in procoagulatory activity and endothelial dysfunction (40). Among these, insulin resistance is most critical as it impairs vascular function, leading to impaired nitric oxide mediated vasorelaxation that contributes to hypertension and to an increased risk of atherosclerosis (41). Consequently, it is not surprising that overweight and obese populations have a lower probability of recovering to normal/elevated BP but a higher probability of progressing from stage 1 to stage 2 hypertension, as insulin resistance may be reversed with regular physical exercise, medication, and weight loss.

Another significant finding in this study relates to the relationship between socio-economic status and the probability of hypertension severity. The results of our study do show that while lower education, rural residence, and lower wealth were associated with a higher probability of progressing to stage 1 hypertension; higher education, urban residence, and higher wealth acquisitions were associated with a higher probability of progressing to a more severe form of hypertension. This is consistent with the findings of Tenkorang and Kuuire (42) on the negative social gradient in health in which Ghanaians with a higher socio-economic status were more likely to live with non-communicable diseases than those with low socio-economic status. At the core of this quandary is the association of overweight and obesity with success, wealth, and good health (43). This false sense of health and well-being creates a condition where an increase in wealth leads to changes in food preference, increase in food consumption, and poor choices with dietary intake (32), culminating in severe hypertension and other cardiometabolic diseases.

Conversely, we provided the needed evidence that shows that an intake of healthy foods such as fruits and vegetables of any quantity per day had the potential of delaying a population's transition from normal/elevated BP to Stage 1 hypertension. However, those who consumed the recommended serving of fruits and vegetables a day had a higher recovery from stage 1 hypertension to normal/elevated BP than those who did not consume the recommended servings, which is an equivalent of 400 grams of fruits and/or vegetables per day. This finding is consistent with existing literature, which prescribes the consumption of more servings of fruits and vegetables per week as a mechanism for delaying or managing severe hypertension (44, 45). Taken singly or together, these sub-groups provide novel start-points for the development and implementation of interventions geared toward hypertension prevention.

The primary strength of this paper is in the use of a longitudinal data which enables a better estimation of progression in disease state than cross-sectional data, which only estimate the prevalence of a disease at a single time point. With the lack of longitudinal data in most SSA countries, the WHO SAGE longitudinal study presents a suitable dataset to estimate such transitions. Second, the use of transition probabilities in estimating progression across hypertension severity states presents a more robust approach to past models which have attempted to predict the likelihood of being in either state at a single time point. Third, this paper provides estimates useful for the calculation of the impact hypertension may have on quality or disability adjusted life years as well as the simulation of cost-effective models to evaluate policies and the burden of future healthcare.

There are some limitations in this study that provide opportunities for future research. First, the WHO SAGE data while ideal, has fewer respondents below the age of 41, which is disproportional to those above 50. Consequently, transitions estimated among the younger cohort should be interpreted with caution as the data may not be representative of the younger cohort in Ghana. Second, our inability to account for the past medical condition of these respondents and the medical treatments received may provide an incomplete picture of disease progression, especially, for those who started at hypertension stage 1 and 2 in 2007/2008. This was due to over 90% of missing data on the intake of anti-hypertensive medication over the period of data collection. However, the Markov assumption that the future of a disease state depends on the current state, and not on the history of the disease state makes our analysis adequate (38). Again, without data on those who received medication after the assessment of their hypertension status in wave 1, it is difficult to estimate the exact time it takes individuals on medications to recover from a worse hypertensive state. Future studies should consider past medical conditions, use of medication, and treatment in estimating transition probabilities. Third, by grouping normal and elevated BP together, we assume that respondents in both categories are in a healthy state, which may not be so for those in the elevated cohort. However, the diastolic threshold for both normal and elevated BP are the same, making it easier to be able to create a common cut-off point in creating the normal/elevated BP state. Lastly, the AHA/ACC recommends that to establish or diagnose hypertension, at least two measurements from two separate visits be conducted before BP status is assigned (10). However, in both waves of this study, BP status was assigned based on a single visit, which may account for misclassification of BP status in wave 1 leading to a possible decline of stage 2 hypertension at wave 2. Future studies may need to diagnose elevated BP based on at least two separate visits to ensure the validity of BP groups.

## Conclusions

This paper contributes new knowledge to the epidemiology of chronic diseases across the life course in four ways. First, this study is the first to measure progression in hypertensive severity in SSA; highlighting the transition probability from normal/elevated BP to stage 1 hypertension or to stage 2 hypertension and the recovery from the latter. Additionally, it shows the importance of having new thresholds for early diagnosis of hypertension. Second, the findings suggest that the consumption of recommended servings of fruits and vegetables per day has the potential to delay the onset of severe hypertension and the recovery from same. Third, the findings in this study can be used in simulating cost-effective measures in the management, control, and treatment of hypertension. Next, the findings provide approximate sojourn and transition times that are adequate in evaluating the performance of an intervention or targeting interventions to specific subgroups with better health outcomes. Finally, the estimates generated from these data are important in calculating the impact hypertension may have on quality/disability-adjusted life years, life expectancy, and the total costs of treating and controlling hypertension within Ghana's population.

## Data Availability Statement

Data for this study were drawn from the World Health Organization Study on global AGEing and adult health (WHO-SAGE). Details of the data can be found at: http://www.who.int/healthinfo/sage/cohorts/en/.

## Ethics Statement

The studies involving human participants were reviewed and approved by WHO Ethics Review Committee (reference number RPC149) with local approval from the University of Ghana Medical School Ethics and Protocol Review Committee (Ghana). The necessary permission was obtained from the World Health Organization to use these data. The participants provided their written informed consent to participate in this study.

## Author Contributions

GB, SL, PB, ZT: conceptualization. GB, SL, RB, PK: data curation. GB, SL, LS, CM: formal analysis. GB, SL, PB, LS, CM: methodology. IL: supervision. GB, SL, PB, LS, RB, PK, CM, ZT, AP, IL: writing—original draft and writing—review and editing. All authors: contributed to the article and approved the submitted version.

## Conflict of Interest

The authors declare that the research was conducted in the absence of any commercial or financial relationships that could be construed as a potential conflict of interest.
