# Supplementary material for: Measuring Hypertension Progression With Transition Probabilities: Estimates From the WHO SAGE Longitudinal Study
Source: Front Public Health. 2021 Apr 7;9:571110. doi: 10.3389/fpubh.2021.571110 (PMC8058215; doi:10.3389/fpubh.2021.571110)
Supplement: Supplementary file 1 [file Data_Sheet_1.docx]

| **Appendix A.** Hazard Ratios from Multistate Markov Model fitted separately for covariates for transitions between normal/elevated blood pressure, Stage 1 and Stage 2 Hypertension across adults (18+ years) in Ghana between 2007/2008 and 2014/2015 | | | | | | | | | |
| --- | --- | --- | --- | --- | --- | --- | --- | --- | --- |
|  | | Hazard ratios (HR) and 95% CI | | | | | | | |
|  |  | From Normal BP to Stage 1 Hypertension | | From Stage 1 Hypertension to Normal BP | | From Stage 1 to Stage 2 Hypertension | | From Stage 2 to Stage 1 Hypertension | |
|  |  | HR | 95% CI | HR | 95% CI | HR | 95% CI | HR | 95% CI |
| Sex | Males | 1.00 | Referent | 1.00 | Referent | 1.00 | Referent | 1.00 | Referent |
|  | Females | 1.03 | 0.61, 1.75 | 1.04 | 0.68, 1.59 | 1.61 | 0.91, 2.85 | 1.11 | 0.72, 1.71 |
| Age group, years | ≤40 | 1.00 | Referent | 1.00 | Referent | 1.00 | Referent | 1.00 | Referent |
|  | 41-60 | 0.48 | 0.04, 5.67 | 0.27 | 0.03, 2.83 | 1.69 | 0.59, 4.85 | 1.36 | 0.67, 2.76 |
|  | >60 | 0.56 | 0.05, 6.56 | 0.30 | 0.03, 3.26 | 1.93 | 0.68, 5.53 | 1.02 | 0.50, 2.07 |
| BMI categories | Underweight | 1.42 | 0.41, 4.87 | 1.45 | 0.47, 4.41 | 0.926 | 0.40, 2.15 | 1.05 | 0.54, 2.04 |
|  | Normal weight | 1.00 | Referent | 1.00 | Referent | 1.00 | Referent | 1.00 | Referent |
|  | Overweight | 1.66 | 0.46, 2.06 | 1.26 | 0.73, 2.19 | 1.77 | 0.80, 3.91 | 1.24 | 0.68, 2.27 |
|  | Obesity | 0.57 | 0.20, 1.61 | 0.77 | 0.42, 1.42 | 1.68 | 0.48, 5.85 | 1.11 | 0.43, 2.88 |
| Educational Status | High | 1.00 | Referent | 1.00 | Referent | 1.00 | Referent | 1.00 | Referent |
|  | Low | 1.59 | 0.92, 2.75 | 1.44 | 0.94, 2.19 | 0.95 | 0.51, 1.77 | 0.85 | 0.53, 1.36 |
| Place of Residence | Rural | 1.00 | Referent | 1.00 | Referent | 1.00 | Referent | 1.00 | Referent |
|  | Urban | 0.90 | 0.52, 1.54 | 0.85 | 0.55, 1.29 | 1.21 | 0.69, 2.13 | 1.03 | 0.67, 1.57 |
| Wealth Index | Lowest | 1.30 | 0.51, 3.33 | 1.09 | 0.47, 2.55 | 0.88 | 0.39, 1.95 | 1.04 | 0.55, 1.95 |
|  | Low | 0.96 | 0.40, 2.28 | 0.89 | 0.43, 1.69 | 0.62 | 0.29, 1.32 | 0.87 | 0.51, 1.49 |
|  | Moderate | 0.86 | 0.37, 2.00 | 0.78 | 0.41, 1.50 | 0.73 | 0.33, 1.63 | 1.10 | 0.63, 1.94 |
|  | High | 1.00 | Referent | 1.00 | Referent | 1.00 | Referent | 1.00 | Referent |
|  | Highest | 0.74 | 0.32, 1.67 | 0.60 | 0.32, 1.14 | 1.78 | 0.57, 5.60 | 1.88 | 0.72, 4.93 |
| Fruit and vegetable intake | Met requirement | 1.00 | Referent | 1.00 | Referent | 1.00 | Referent | 1.00 | Referent |
|  | Below requirement | 0.84 | 0.48, 1.49 | 1.09 | 0.70, 1.69 | 1.06 | 0.59, 1.91 | 1.09 | 0.71, 1.68 |
| Notes: Significant result at *P*< 0.05. | | | | | | | | | |
